# Role of Rip2 in Development of Tumor-Infiltrating MDSCs and Bladder Cancer Metastasis

**DOI:** 10.1371/journal.pone.0094793

**Published:** 2014-04-14

**Authors:** Hanwei Zhang, Arnold I. Chin

**Affiliations:** Department of Urology, Broad Stem Cell Research Center, Jonsson Comprehensive Cancer Center, University of California Los Angeles, Los Angeles, California, United States of America; University of Alabama at Birmingham, United States of America

## Abstract

Tumor invasion and metastases represent a complex series of molecular events that portends a poor prognosis. The contribution of inflammatory pathways mediating this process is not well understood. Nod-like receptors (NLRs) of innate immunity function as intracellular sensors of pathogen motifs and danger molecules. We propose a role of NLRs in tumor surveillance and in programming tumor-infiltrating lymphocytes (TILs). In this study, we examined the downstream serine/threonine and tyrosine kinase Rip2 in a murine model of bladder cancer. In Rip2-deficient C57Bl6 mice, larger orthotopic MB49 tumors developed with more numerous and higher incidence of metastases compared to wild-type controls. As such, increased tumor infiltration of CD11b^+^Gr1^hi^ myeloid-derived suppressor cells (MDSCs) with concomitant decrease in T cells and NK cells were observed in Rip2-deficient tumor bearing animals using orthotopic and subcutaneous tumor models. Rip2-deficient tumors showed enhanced epithelial-to-mesenchymal transition, with elevated expression of *zeb1*, *zeb2*, *twist*, and *snail* in the tumor microenvironment. We found that the absence of Rip2 plays an intrinsic role in fostering the development of granulocytic MDSCs by an autocrine and paracrine effect of granulocytic colony stimulating factor (G-CSF) expression. Our findings suggest that NLR pathways may be a novel modality to program TILs and influence tumor metastases.

## Introduction

The context of the immune tumor microenvironment can be predictive of tumor stage, cancer-specific survival, as well as response to chemotherapy [1]–[3]. In certain malignancies, the presence of infiltrating CD8^+^ T lymphocytes correlates with improved outcomes, whereas infiltrating B cells, CD4^+^ T lymphocytes, and negative regulators including myeloid-derived suppression cells (MDSCs) and T regulatory cells (Tregs) negatively correlate with survival [4]. These observations underscore the importance in defining pathways essential to programming the composition of tumor infiltrating lymphocytes (TILs), as selective modulation of the immune tumor microenvironment represents an emerging therapeutic modality.

Pattern recognition receptors such as the prototypic Toll-like receptor (TLR) family and intracellular nucleotide-binding oligomerization domain (NOD)-like receptor (NLR) family recognize conserved motifs on pathogens as well as endogenous molecules released by damaged cells [5]. Activation of these signaling pathways initiates innate and adaptive immune responses, and may promote tissue repair and regeneration. We previously implicated TLR3 in a murine model of prostate cancer tumor surveillance critical in programming the infiltration of T lymphocytes and NK cells, while suppressing Treg expansion [6]. In bladder cancer, the efficacy of immune modulation is highlighted by the use of intravesical Bacillus Calmette Guerin, which promotes an influx of macrophages and neutrophils within the tumor microenvironment mediated in part by TLR2 and 4 [7], [8].

Receptor-interacting protein 2 (Rip2), a serine-threonine and tyrosine kinase downstream and common to Nod1 and Nod2, activates transcription factors such as NF-κB and MAP kinases through its kinase activity as well as through recruitment of E3 ubiquitin ligase [9]–[14]. Rip2-dependent pathways have emerged as critical in sensing diverse pathogens ranging from *Listeria monocytogenes*, *Staphylococcus aureus*, and *Legionella pneumophilia* to *Escherichia coli* as well as in mediating inflammatory disorders such as autoimmune encephalomyelitis [15]–[17]. In addition, polymorphisms of Nod2 have been linked with susceptibility to Crohn's disease, while polymorphisms of Rip2 have been linked with systemic lupus erythematosus [18], [19]. The ability of NLRs to mediate tumor surveillance has not been investigated to date. Here, we postulate that Rip2 may mediate bladder cancer surveillance and explore its role using a murine orthotopic and subcutaneous bladder cancer model. We show that tumor bearing Rip2-deficient mice biases myeloid differentiation towards the MDSC lineage and plays an intrinsic role in the development of the CD11b^+^Ly6G^+^Ly6C^lo^ granulocytic MDSC population. Our findings are the first to implicate Rip2 and NLRs in tumor surveillance and their importance in programming the immune tumor microenvironment. The NLR pathway may represent a therapeutic opportunity in modulating cancer immunity to prevent tumor invasion and metastasis.

## Materials and Methods

### Ethics Statement

All animal work has been conducted in accordance with the Public Health Service Policy on Human Care and Use of Laboratory Animals and USDA Animal Welfare Act Regulations through an approved UCLA Institutional Animal Care and Use Committee protocol #2010-023-11C.

### Mice

Rip2-deficient mice backcrossed to a C57Bl/6 background for 10 generations were genotyped as previously described [10]. Age-matched C57Bl/6 mice (Jackson Laboratories) were used as controls. Six- to 8-wk old female mice were used for the experiments. Mice were housed in pathogen-free conditions according to UCLA Animal Research Committee protocols.

### Cell culture

MB49 cell lines (gift from Tim Ratliff) were derived from carcinogen-induced urothelial cell carcinomas in C57Bl6 mice and maintained at 37°C with 5% CO_2_ in DMEM (Cellgro), supplemented with 10% FBS (Omega Scientific) and 1% penicillin and streptomycin [20].

### Tumor models

For intravesical tumor implantation, female mice were sedated, catheterized with a 24 gauge catheter (BD Biosciences), and instilled with 10 µg of poly-L-lysine (Sigma) in 100 µl for 30 minutes before drainage and instillation of 2×10^6^ MB49 cells in 100 µl for 60 minutes [21], [22]. Mice were sacrificed 12 days following tumor inoculation, and the bladder and internal organs were examined. Bladder, lungs, and kidneys were fixed in formalin or embedded in OCT for staining by H&E or immunofluorescence [23]. Pulmonary and renal metastases were determined grossly and by light microscopy of H&E sections in a single representative coronal cross section. For subcutaneous tumor challenges, 5×10^5^ MB49 cells were implanted subcutaneously in the flanks of mice with tumor growth monitored every other day. Following sacrifice of the mice 14 days following tumor inoculation, tumors were harvested, weighed, and dissociated for analysis.

### Immunofluorescence and Immunohistochemistry

Immunofluorescence was performed on OCT-embedded frozen tissue sections. Sections were blocked with 10% goat serum and 10% BSA in PBS for 1 hour at RT, incubated with primary antibody overnight at 4°C, then washed and incubated with Al-448 or Al-466 goat anti-rat secondary antibody (Invitrogen) at 1∶800 dilution. Immunohistochemistry was performed on formalin-fixed paraffin-embedded tumor sections. Sections were subject to heated antigen unmasking solution for 30 minutes, quenched in 0.3% hydrogen dioxide in PBS for 15 minutes, blocked with 10% goat serum and 10% BSA in PBS for 1 hour at RT, then incubated with primary antibody overnight at 4°C, incubated with biotinylated goat anti-rat secondary antibody at 1∶800 dilution for 30 minutes at RT, and developed using the ABC kit (Vector Labs). Slides were counterstained with hematoxylin. Sections were mounted with Vectorshield and analyzed by fluorescence or light microscopy (Zeiss). Antibodies include CD11b (M1/70, R&D Systems), CD4 (RM4–5, Biolegend), CD8 (53.6–7, R&D Systems), CD49b (DX-5, Biolegend), Foxp3 (FJK-16s, eBioscience), Ly6G (RB6-BC5, eBioscience), Ly6C (AL-21, BD Biosciences), E-Cadherin (MAB7481, R&D Systems), N-cadherin (H-63, Santa Cruz Biotechnology), and phospho-IκBα (Ser 32/36, #9246, Cell Signaling).

### Cytokine array

An inflammatory cytokine array comparing serum from wild-type and Rip2-deficient mice implanted with intravesical MB49 cells was performed as described (ARY006, R&D Systems).

### Flow cytometry analysis

Single-cell suspensions prepared from spleens, dissociated tumor cells, or differentiated bone marrow cells were analyzed by flow cytometry. Antibodies including CD4 (RM-4), CD8 (53–67), NK1.1 (pk-136), B220 (RA3-6B2), CD11b (M1/70), CD45.2 (104), Gr1 (RB6-8C5), Ly-6G (1A8), and Ly-6C (AL21) were obtained from BD Biosciences. Data acquisition was performed on a FACS LSRII (BD Biosciences) and analyzed using FlowJo.

### Bone marrow-derived myeloid differentiation

BM-derived cells were obtained by flushing femurs and tibia from C57Bl/6 wild-type and RIP2^−/−^ mice. Following lysis of red blood cells with ACK buffer, the remaining cells were cultured in DMEM supplemented with 10% FBS and 20% L929 cell-conditioned media. After incubation for 24 hours, non-adherent cells were harvested and plated at 2×10^5^ cells/mL in DMEM supplemented with 10% fetal calf serum, 50 µM 2-mercaptoethanol, 10 mM HEPES, 1 mM sodium pyruvate, 1% penicillin and streptomycin, and supplemented using 20% L929 with 40 ng/mL G-CSF (Biolegend), 40 ng/mL GM-CSF (Invitrogen), or in combination. Cultures were incubated at 37°C with 5% CO_2_ for 3 days. On Day 3, fresh medium with appropriate cytokines was added. On Day 4 cultured cells were harvested for FACS.

### Isolation of tumor-infiltrating immune cells

Tumor tissue was minced in PBS with sterile scissors to approximately 1 mm pieces and digested in 0.25% collagenase IV in DMEM supplemented with 10% FBS at 37°C with 5% CO_2_ for 3 hours [24]. Cell suspensions were filtered through a 70 µm cell strainer, subjected to ACK buffer for 5 minutes on ice, and then filtered through a 20 µM cell strainer for flow cytometry. Cells sorted using a FACS Aria (BD Biosciences) into CD45^+^CD11b^+^Gr1^hi^, CD45^+^CD11b^+^GR1^lo^, and CD45^−^ fractions were collected for qPCR.

### Quantitative RT-PCR

Total RNA extracted from sorted cells and fresh frozen bladder tumor as described (RNeasy Mini Kit, Qiagen) was used to synthesize cDNA using High Capacity cDNA Reverse Transcription Kits (Applied Biosystems). Relative gene expression was determined using SYBR Green PCR Master Mix (Applied Biosystems). A Bio-Rad iCycler was used to analyze the samples while the comparative threshold cycle method was used to calculate gene expression normalized to GAPDH as a gene reference. Primers sets for the following genes were used: Arginase-1, 5′-AGAGATACTTCCAACTGCCAGACT, 3′-ACCTGGCCTTTGTTGATGTCCCTA; iNOS, 5′-GCTGGAAGCCACTGACACTTCG, 3′-CGAGATGGTCAGGGTCCCCT; IL-4, 5′- GTCATCCTGCTCTTCTTTC, 3′- ATGGCGTCCCTTCTCCTGT; IL-10, 5′-GCTCTTACTGACTGGCATGAG, 3′-CGCAGCTCTAGGAGCATGTG; IL-12 p40, 5′- TGGTTTGCCATCGTTTTGCTG, 3′- ACAGGTGAGGTTCACTGTTT; IFNγ, 5′- GGATGCATTCATGAGTATTGC, 3′- CCTTTTCCGCTTCCTGAGG; M-CSF, 5′- AGGACCTGTTGGAGTTCCCTC, 3′- TTTCGCCCTCACACTTGATGA; G-CSF, 5′- CTCAACTTTCTGCCCAGAGG, 3′- AGCTGGCTTAGGCACTGTGT; GM-CSF, 5′- GCCATCAAAGAAGCCCTGAA, 3′- GCGGGTCTGCACACATGTTA; Zeb1, 5′-AACGGAGATTTGTCTCCCAGT, 3′-CTGTCCAGCTTGCATCTTTTC; Zeb2, 5′-TAGCCGGTCCAGAAGAAATG, 3′-GGCCATCTCTTTCCTCCAGT; Snail, 5′-GCGGAAGATCTTCAACTGCAAATATTGTAAC, 3′-GCAGTGGGAGCAGGAGAATGGCTTCTCAC; Twist, 5′-CGGGTCATGGCTAACGTG, 3′-CAGCTTGCCATCTTGGAGTC; and GAPDH, 5′-GACCCCTTCATTGACCTCAAC, 3′-CTTCTCCATGGTGGTGAAGA.

## Results

**Figure 1:**
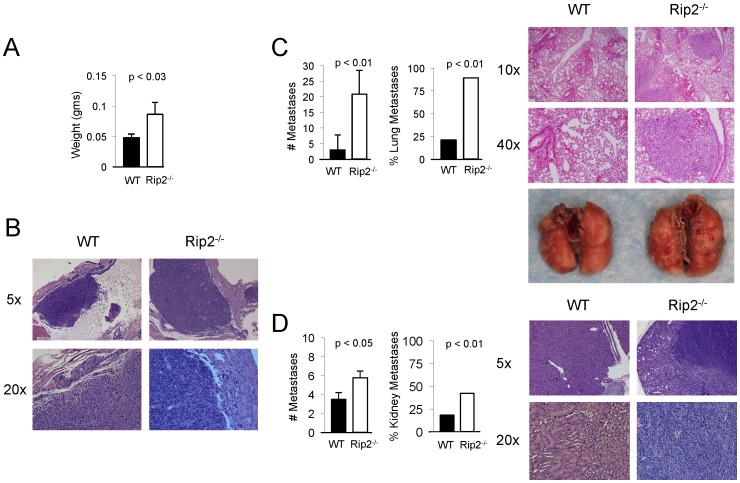
Increased tumor size and metastases in Rip2-deficient mice. Rip2^+/+^ and Rip2^−/−^ mice intravesically implanted with MB49 cells and sacrificed at 12 days were assessed for bladder tumor (A) weight, (B) histology with H&E staining at x5 and x20 magnification. C, Lungs were examined for metastasis by number of metastatic lesions per coronal cross section, % incidence, histology with H&E staining at x10 and x40 magnification, and gross examination. D, Kidneys were examined for metastasis by number of metastatic lesions per coronal cross section, % incidence, and histology with H&E staining at x5 and x20 magnification. Representative examples are shown. Columns, mean of 33 Rip2^+/+^ and 19 Rip2^−/−^ mice pooled from 5 independent experiments; bars, SEM. All *p* values were determined by two-tailed Student's *t* test, with statistically significance defined as p<0.05.

To investigate the role of Rip2 in tumor surveillance, we challenged wild-type and Rip2-deficient mice with syngeneic MB49 cells in an orthotopic bladder cancer model. We observed an almost two-fold increase in tumor weight in the absence of Rip2 (Fig 1A). Tumors harvested from mice of both genotypes demonstrated evidence of invasion into the detrusor muscle by H&E staining under light microscopy with larger tumors seen in Rip2-deficient animals (Fig 1B). Gross and histologic examination of internal organs revealed a greater number of metastasis in the lungs and kidneys of Rip2-deficient mice at an approximately three-fold and two-fold higher incidence respectively over wild-type controls (Fig 1C–D). No other gross metastases were observed including in the liver.

**Figure 2:**
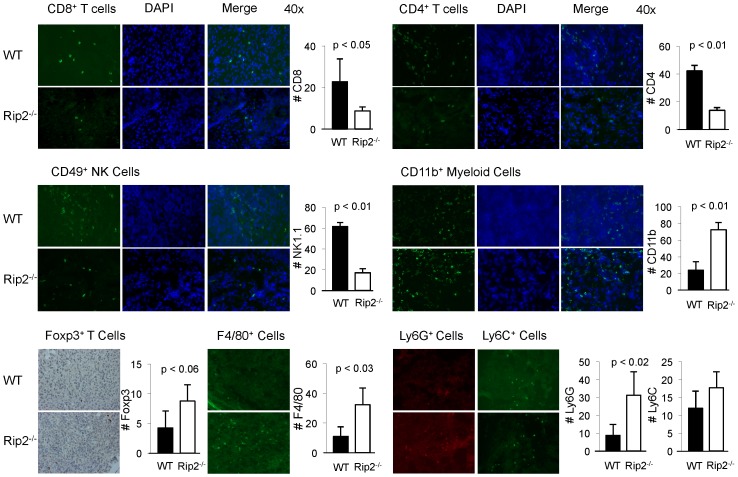
Loss of Rip2 alters composition of tumor infiltrating cells from Rip2-deficient mice. Infiltration of CD8, CD4, NK, CD11b, F4/80, Ly6G, and Ly6C expressing cells as indicated, were examined by immunofluorescence, and Foxp3 by immunohistochemistry in bladder tumors from Rip2^+/+^ and Rip2^−/−^ mice intravesically implanted with MB49 cells and sacrificed at 12 days. Left panels show specific antibody staining, middle panel shows DAPI staining, right panel shows merging of antibody and DAPI stains. Representative examples from each group of four to six mice in 4 independent experiments are shown at x40 magnification. Bar graphs enumerate mean number of cells per x40 field of 3 representative sections from groups of four mice; bars, SD. All *p* values were determined by two-tailed Student's *t* test, with statistically significance defined as p<0.05.

We have previously implicated Rip2 in development of Th1 and NK cells [10], while others reported a role in dendritic cell infiltration [17]. To investigate the role of Rip2 in the context of tumorigenesis, bladder tumor TILs were examined by immunofluorescence and immunohistochemistry. This revealed decreased infiltration of CD8^+^ T lymphocytes, CD4^+^ T lymphocytes, and NK cells in the absence of Rip2 as predicted. Examination of Foxp3^+^ cells representative of T regulatory cells, showed an increasing trend in Rip2-deficient mice compared to wild-type mice although it did not reach statistical significance. Interestingly, tumors showed increased infiltration of CD11b^+^ myeloid cells in Rip2-deficient compared to wild-type mice, while additional characterization of this myeloid population showed an expected increase in the macrophage marker F4/80 and in the Gr-1 subtype Ly6G, but not subtype Ly6C in tumors from Rip2-deficient mice (Fig 2).

**Figure 3:**
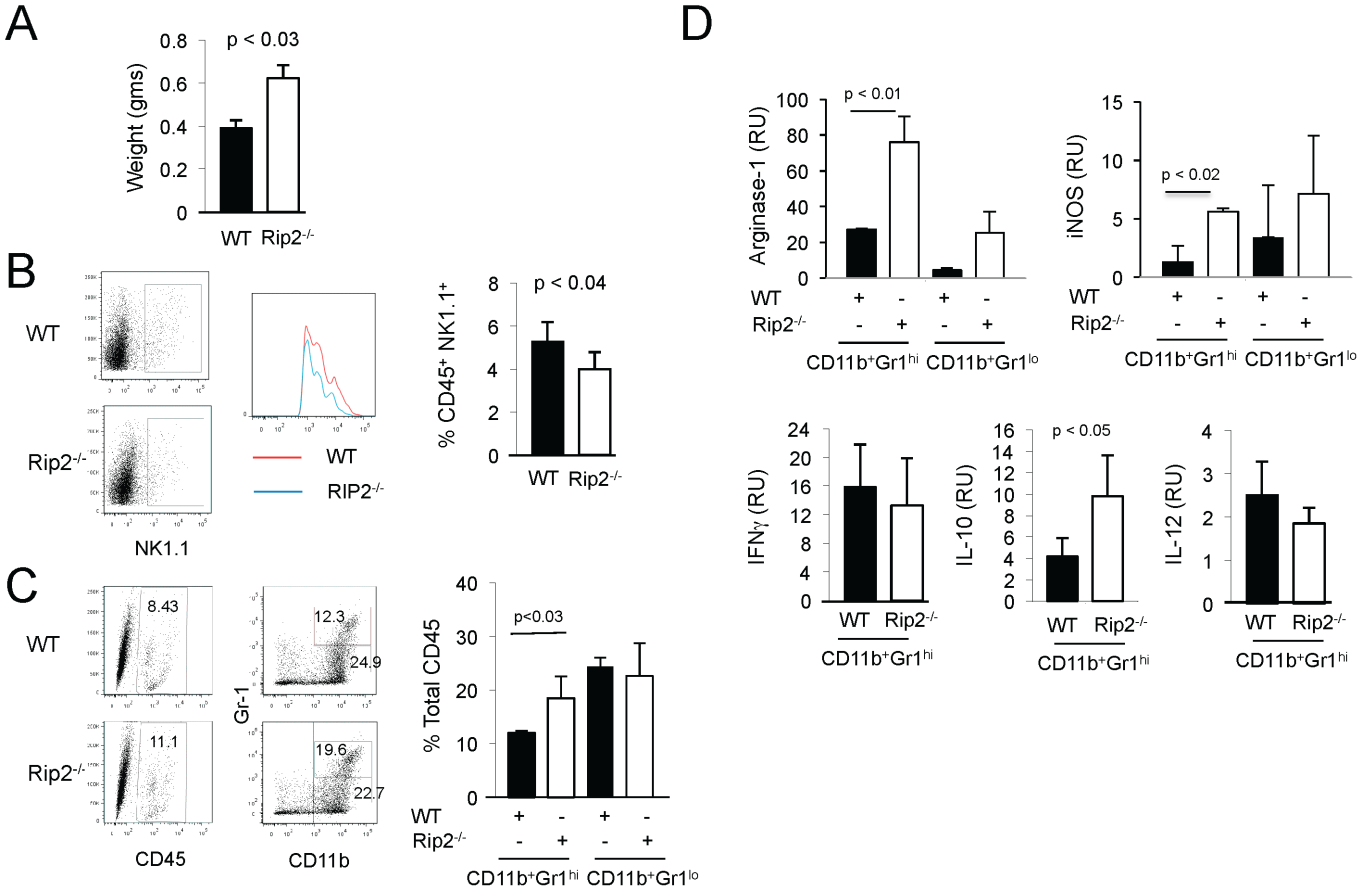
Increased tumor infiltrating granulocytic MDSCs in Rip2-deficient mice. Rip2^+/+^ and Rip2^−/−^ mice subcutaneously implanted with MB49 cells for 14 days were assessed for (A) tumor weight, and sorted by flow cytometry to examine (B) CD45^+^NK1.1^+^ cells, and (C) CD45^+^CD11b^+^Gr1^hi^ and CD45^+^CD11b^+^Gr1^lo^ cells. Column, mean of four mice; bars SD. Data are representative of two independent experiments. D, Sorted CD45^+^CD11b^+^Gr1^hi^ and CD45^+^CD11b^+^Gr1^lo^ cells in Rip2^+/+^ and Rip2^−/−^ mice were examined for expression of arginase-1 and iNOS by qPCR, while CD45^+^CD11b^+^Gr1^hi^ cells in Rip2^+/+^ and Rip2^−/−^ mice were examined for expression of IL10, IL-12, and IFNγ by qPCR. Column, mean of two mice; bars SD. Data are representative of three independent experiments. Representative examples are shown. All *p* values were determined by two-tailed Student's *t* test, with statistically significance defined as p<0.05.

To further examine the function of the tumor infiltrating populations, we subcutaneously implanted MB49 cells to generate larger tumors to facilitate subsequent sorting of cells. Similarly to orthotopic tumors, subcutaneous tumors developed in Rip2-deficient mice were of increased size compared to wild-type counterparts (Fig 3A). Dissociated tumors sorted for CD45^+^NK1.1^+^ NK cells showed decreased numbers in Rip2-deficient compared to wild-type mice as expected (Fig 3B). The infiltrating numbers of CD4 and CD8 cells were too small to quantitate by flow cytometry (data not shown). Consistent with the previous orthotopic tumors, dissociated subcutaneous tumors sorted for surface markers CD11b^+^ and Gr1^+^ showed enriched numbers of CD11b^+^Gr1^hi^ cells from Rip2-deficient compared to wild-type mice (Fig 3C). To further examine the functional characteristics of the CD11b^+^Gr1^hi^ cells, we examined a panel of genes that represent a signature of MDSCs and myeloid subpopulations. Arginase-1 and iNOS mediate the T suppressive effects of MDSCs [25]. IFNγ supports the differentiation towards M1 cells, distinguished in turn by secretion of IL-12. IL-4 polarizes myeloid precursors towards a M2 phenotype, which is strongly associated with tumor associated MDSCs and express IL-10 to mediate their immune suppressive effects [26]. We show that Rip2-deficient CD11b^+^Gr1^hi^ cells expressed higher levels of arginase-1 compared to cells from wild-type mice, with a similar trend in iNOS expression (Fig 3D). Consistently, increased IL-10 expression in Rip2-deficient CD11b^+^Gr1^hi^ cells compared to cells from wild-type controls were observed, while no significant differences were seen in expression of IFNγ and IL-12, indicative of M2 polarization (Fig 3D). Levels of IL-4 were undetectable in the CD11b^+^Gr1^hi^ cells (data not shown).

**Figure 4:**
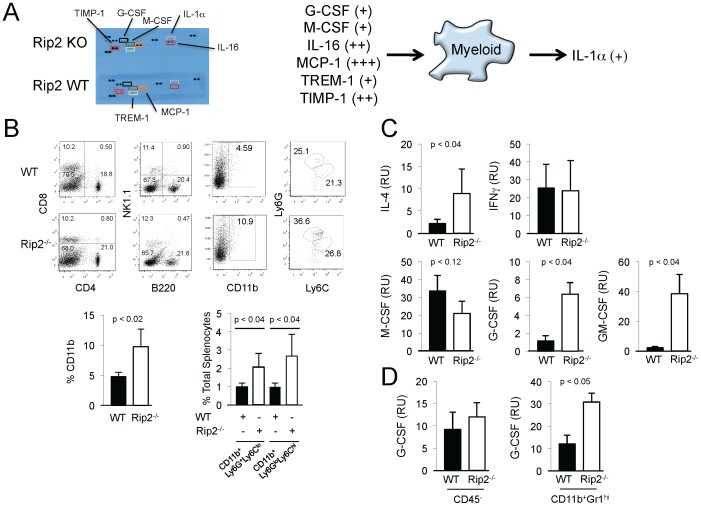
Biased development of granulocytic MDSCs in Rip2-deficient mice. A, Inflammatory cytokine expression in serum of Rip2^+/+^ and Rip2^−/−^ mice intravesically implanted with MB49 cells for 12 days was assessed by cytokine array analysis. Cytokines increased in Rip2^−/−^ mice are depicted in the schematic as shown. (+) 2-5x, (++) 5-10x, (+++) >10x based on quantitation by ImageJ. Results are from duplicates and representative of two independent experiments. B, Splenocytes from Rip2^+/+^ and Rip2^−/−^ mice were examined for expression of CD4, CD8, B220, NK1.1, while CD11b^+^ cells were examined for expression of Ly6G and Ly6C by flow cytometry as indicated. Column, mean of four mice; bars SD. Data are representative of two independent experiments. C, Total tumor from Rip2^+/+^ and Rip2^−/−^ mice intravesically implanted with MB49 cells and sacrificed at 12 days were examined for expression of IL4, IFNγ, M-CSF, G-CSF, and GM-CSF by qPCR. Column, mean of three mice; bars SD. D, Tumors from Rip2^+/+^ and Rip2^−/−^ mice subcutaneously implanted with MB49 cells for 14 days were fractioned to CD45^−^ and CD45^+^CD11b^+^Gr1^hi^ cells and examined for G-CSF expression by qPCR. Column, mean of three mice; bars SD. All *p* values were determined by two-tailed Student's *t* test, with statistically significance defined as p<0.05.

To examine the Rip-2-dependent systemic cues that may influence the development of the tumor microenvironment, cytokine levels were measured using an inflammatory cytokine array comparing serum from wild-type and Rip2-deficient mice implanted with orthotopic MB49 bladder tumors. The levels of seven cytokines from the panel comprising 40 cytokines were found elevated in the absence of Rip2. The majority were associated with myeloid differentiation and chemotaxis, including G-CSF, M-CSF, IL-16, MCP-1, TREM-1, TIMP-1, and IL-1α with relative levels shown schematically [27]–[33] (Fig 4A). Splenocytes from tumor bearing mice were analyzed to assess the influence of these cytokine alterations, revealing no significant differences in the numbers of CD4^+^ and CD8^+^ T lymphocytes, B220^+^ B lymphocytes, and NK1.1^+^ cells, but increased development of CD11b^+^Ly6G^hi^ cells in Rip2-deficient compared to wild-type mice, that represent the granulocytic MDSC population (Fig 4B). To further examine the bladder tumor microenvironment, we examined a panel of cytokines that influence the functional polarization of macrophages as well as differentiation of MDSCs in the intravesical bladder tumors [34]. Increased levels of IL-4, G-CSF, and GM-CSF were observed in bladder tumors from Rip2-deficient compared to wild-type mice, while no differences were detected in IFNγ and M-CSF expression, consistent with a tumor microenvironment fostering development of the tumor associated MDSCs (Fig 4C). To discriminate between cytokine production from tumor and surrounding stroma to the myeloid populations, we examined G-CSF expression in the CD45^−^ population representing tumor and stromal cells, and CD11b^+^Gr1^hi^ population representing myeloid cells, and showed increased G-CSF in Rip2-deficient compared to wild-type CD11b^+^Gr1^hi^ cells, suggesting an autocrine and paracrine regulation of MDSCs in absence of Rip2 (Fig 4D).

**Figure 5:**
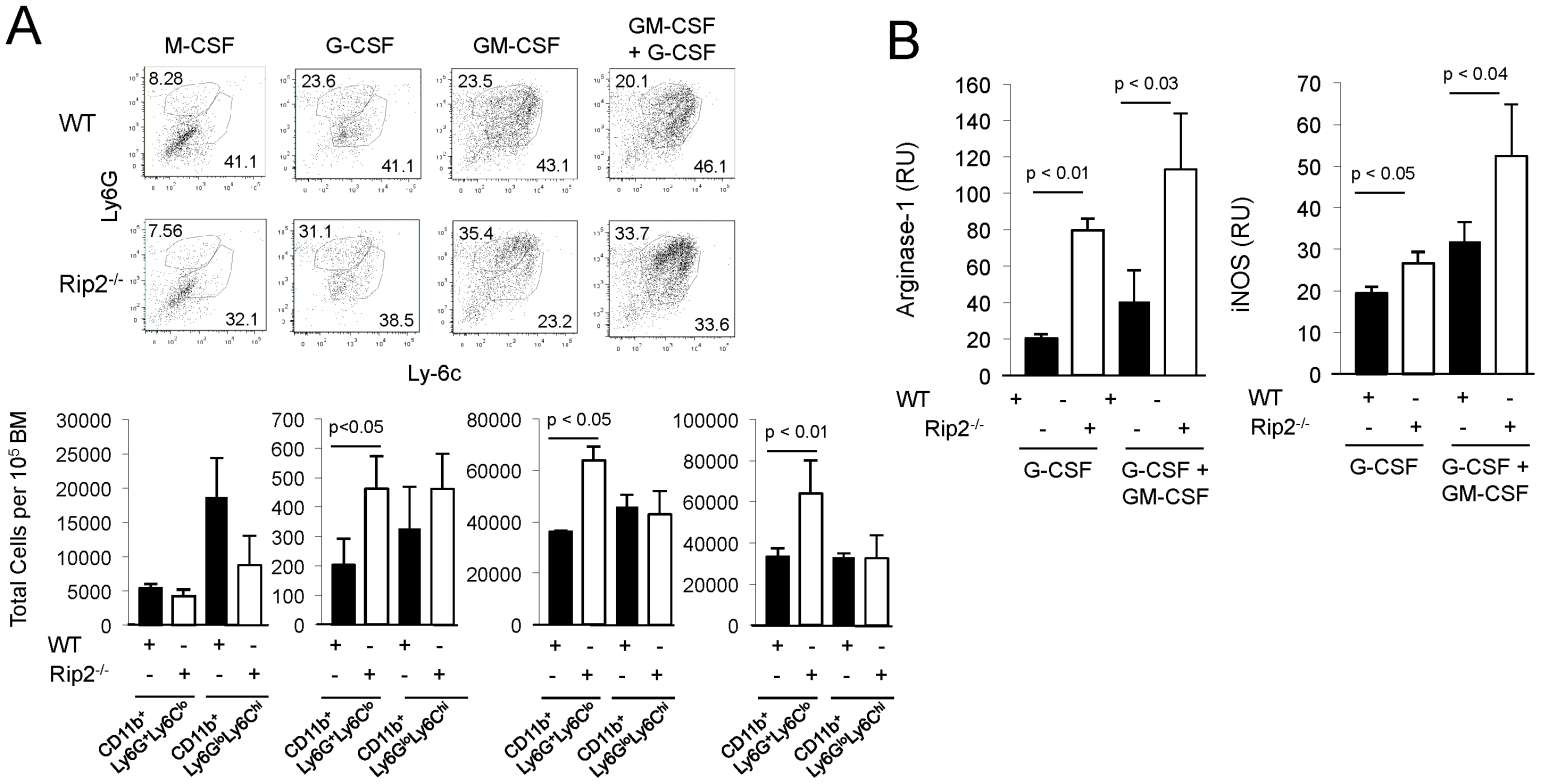
Intrinsic enhanced development of MDSC in absence of Rip2. A, Bone marrow from naïve Rip2^+/+^ and Rip2^−/−^ mice were differentiated by M-CSF, G-CSF, GM-CSF, and G-CSF plus GM-CSF as indicated for four days prior to flow cytometry to examine expression of Ly6G and Ly6C in CD11b^+^ cells (ranging from 70–90%, not shown). CD11b^+^LyG^+^Ly6C^lo^ and CD11b^+^LyG^lo^Ly6C^hi^ populations for each group are shown as total cell counts per 10^5^ starting BM cells. B, Expression of arginase-1 and iNOS by qPCR in G-CSF and G-CSF plus GM-CSF differentiated bone marrow from naïve Rip2^+/+^ and Rip2^−/−^ mice. Column, mean of three mice; bars SD. Data are representative of two independent experiments. Representative examples are shown. All *p* values were determined by two-tailed Student's *t* test, with statistically significance defined as p<0.05.

The MDSC population is comprised of immature myeloid cells and myeloid cell progenitors that can differentiate into mature macrophages, granulocytes, and dendritic cells. They are characterized by T lymphocyte suppressive ability and expression of arginase-1 and iNOS [25]. In the mouse, they can be further distinguished into granulocytic MDSCs by expression of surface markers CD11b^+^Gr1^hi^ or CD11b^+^Ly6G^+^Ly6C^lo^ and monocytic MDSCs by expression of markers CD11b^+^Gr1^lo^ or CD11b^+^Ly6G^lo^Ly6C^hi^ [35]. To test the intrinsic role of Rip2 in myeloid development, naïve bone marrow from wild-type and Rip2-deficient mice were subject to *in vitro* differentiation using M-CSF, G-CSF, GM-CSF, or G-CSF and GM-CSF for 4 days and examined for expression of CD11b, Ly6G, and Ly6C. In the presence of G-CSF, GM-CSF, and G-CSF plus GM-CSF, the absence of Rip2 resulted in a bias towards the CD11b^+^Ly6G^+^Ly6C^lo^ granulocytic MDSC population (Fig 5A). In BM-derived MDSCs differentiated using G-CSF or G-CSF and GM-CSF, increased arginase-1 and iNOS expression was uniformly observed in Rip2-deficient compared to wild-type cells, consistent with the increased development of MDSCs (Fig 5B).

**Figure 6:**
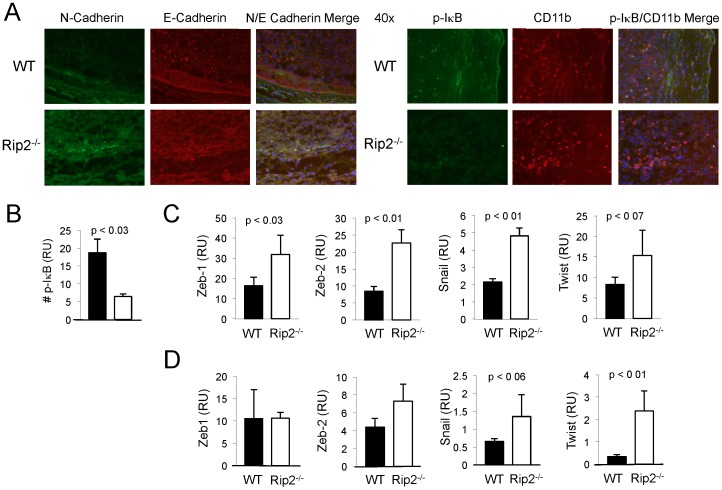
Enhanced epithelial-to-mesenchymal transition in tumors from Rip2-deficient mice. A, Rip2^+/+^ and Rip2^−/−^ mice intravesically implanted with MB49 cells for 12 days were assessed for E-cadherin and N-cadherin expression by immunofluorescence. Magnification of x40. Data are representative of two independent experiments. B, Bar graphs below enumerate mean number of cells per x40 field of 3 representative sections from groups of four mice; bars, SD. C, Expression of *zeb-1*, *zeb-2*, *snail*, and *twist* were examined from CD45^−^ dissociated total tumor tissue by qPCR. Column, mean of four mice; bars SD. Data are representative of two independent experiments. D, Expression of *zeb-1*, *zeb-2*, *snail*, and *twist* were examined from dissociated lungs containing metastases by qPCR. Column, mean of four mice; bars SD. All *p* values were determined by two-tailed Student's *t* test, with statistically significance defined as p<0.05.

MDSCs have been implicated in promoting EMT, a process critical in facilitating tumor invasion and subsequent tumor metastases [36]. A hallmark of EMT is a switch in surface adhesion markers with the loss of E-cadherin and expression of N-cadherin [37]. To investigate the influence of Rip2 in development of EMT, we examined expression of adhesion markers in intravesical tumors and observed decreased E-cadherin expression throughout the tumor with concomitant increased N-cadherin expression at the peripheral of the tumor in Rip2-deficient relative to wild-type mice (Fig 6A). NF-κB activation has been implicated in promoting EMT and thus we examined its activation in the absence of Rip2 [38]. Consistent with prior literature, we observed decreased canonical NF-κB activation exhibited by decreased p-IκB expression in Rip2-deficient tumors (Fig 6B), suggesting NF-κB independent mechanisms to account for EMT [39]. To investigate other mechanisms promoting EMT, a panel of transcription factors involved in EMT including *zeb-1*, *zeb-2*, *snail*, and *twist* were examined in tumor and stromal cells from dissociated subcutaneous tumors depleted of CD45, with the expression of *zeb-1*, *zeb-2*, and *snail* found increased in Rip2-deficient compared to wild-type tumor bearing mice, and increased expression of *twist* approaching statistical significance (Fig 6C). We also examined expression of these EMT inducing genes in lungs containing metastases from Rip2 wild-type and –deficient mice, revealing a similar trend with statistical significance for increased *twist* expression in absence of Rip2 (Fig 6D).

## Discussion

The immune composition of the tumor microenvironment can be predictive of disease-free and overall survival. This has been shown in the context of CD4^+^ T lymphocytes and CD1α dendritic cells in axillary lymph nodes correlating with disease-free survival in breast cancer patients [40], to tumor-infiltrating CD3^+^ cells and CD8^+^ T lymphocytes in colon [1] and bladder cancer respectively [2]. The balance of TILs may refine the predictive ability of the immune microenvironment, as CD4^l^°CD48^l^°CD8^hi^ patients exhibited improved recurrence-free survival while the ratio of CD8^+^ T cells to CD68^+^ tumor associated macrophages predicted overall survival and response to neoadjuvant chemotherapy in breast cancer patients [3]. Anti-tumor immunity may be augmented by targeting negative regulators of T cell co-stimulation as evidenced by the development of anti-CTLA4 and anti-PD1 therapeutics, and may be extended to targeting Tregs and MDSCs [41]. This evidence suggests that active programming of the tumor immune microenvironment may be a viable strategy to harness and bias immunity towards an anti-tumor response.

We have hypothesized that the ability of PRRs to sense contextual signals from pathogens to initiate an immune response is paralleled by the ability of DAMPs released by cellular damage in the tumor environment to shape tumor immunity. NLRs including Nod1 and Nod2 have been implicated in sensing various pathogens as well as cellular stresses [42]. The role of cytosolic PRRs, such as the NLR family in tumor surveillance is unclear. Recently, loss of Rip2 has been implicated in development of larger colorectal cancers in a murine model [43]. In this manuscript, we show that NLRs through Rip2 signaling can shape the tumor microenvironment towards tumor-infiltrating CD8^+^ T lymphocytes, NK cells, and suppression of MDSCs. Similarly, in the absence of Rip2, larger orthotopic bladder tumors developed as well as increased tumor metastases in both the lung and kidneys.

Rip2 was initially described based on sequence homology to its C-terminal CARD domain [44]. We developed Rip2-deficient animals and described a role in innate immunity implicating Rip2 downstream of Nod1, and adaptive immunity mediating IFNγ responses in Th1 and NK development [10]. Since then, the importance of Rip2 in NLR signaling and macrophage function has been validated and shown to play a critical role in innate immune signal transduction of Nod1 and Nod2 receptors [45]. In the absence of tumor growth, we found no significant differences in the development of all splenocytic lineages examined including CD11b^+^Gr1^+^ cells in Rip2-deficient compared to wild-type mice. However, in the presence of tumor growth we observed increased splenocytic CD11b^+^Ly6G^+^ cells representing granulocytic MDSCs in the absence of Rip2 [35].

We examined the systemic macroenvironment as well as local microenvironment levels of cytokines that were altered by the absence of Rip2 expression. Interestingly, elevated serum cytokines important in myeloid differentiation were found in Rip2-deficient tumor bearing animals, with increased levels of G-CSF and M-CSF, as well as MCP-1 and TREM-1. MCP-1 has been implicated in higher recurrence and worse bladder cancer prognosis by mediating tumor invasion, while TREM-1 has been implicated in activation of Kuffner cells in hepatocellular carcinoma [27], [30]. Within the tumor microenvironment, in the absence of Rip2, we found a cytokine signature enriched in IL-4 and G-CSF, polarizing towards a M2 phenotype as reflected in increased production of IL-10 and arginase-1 [46]. M2 macrophages have been associated with tumor progression as well as MDSC development [47].

Examining the tumor infiltration population, we found not only increased proportion of CD11b^+^Gr1^hi^ cells in Rip2-deficient animals, but also increased expression of arginase-1 and iNOS, which leads to inhibition of T cells and NK cells, and suggests an intrinsic function of Rip2 in MDSC development. Rip2-deficient CD11b^+^Gr1^hi^ MDSCs produced elevated levels of IL-10 which may further inhibit T cell activity by enhancing Tregs [48]. With the increased activation of Rip2-deficient MDSCs, we asked whether Rip2 plays an intrinsic role in the development of CD11b^+^ subsets. Bone marrow-derived MDSCs using various cytokines showed increased development of CD11b^+^Ly6G^+^Ly6C^lo^ granulocytic MDSCs in the presence of G-CSF, GM-CSF, or their combination in Rip2-deficient compared to wild type cells, while there was no increased development of monocytic MDSC. The increased serum production of G-CSF as well as an intrinsic development of granulocytic MDSCs *in vitro* in response to G-CSF, suggests a mechanism for increased granulocytic MDSCs in the absence of Rip2.

Growing evidence has attributed MDSCs in cancer progression to immune suppression and modifying the microenvironment to foster tumor metastasis. Increased MDSCs have been described in cancer patients, including patients with bladder cancer [49], [50]. Granulocytic MDSCs predominate in TIL populations and suppress CD8+T cells by producing reactive oxidative species (ROS), Arg1, and iNOS [25], [35]. Recent studies support the role of granulocytic MDSCs to induce EMT in a melanoma model mediated by the chemokine receptor expressed on MDSCs, CXCR2 [36]. During metastasis, it is essential that cancer cells acquire a motile phenotype that allows for tumor invasion, extravasation into the blood stream or lymphatic channels for dissemination, followed by tumor implantation and growth. We noted increased tumor infiltrating MDSCs, decreased T lymphocytes and NK cells, and larger tumors with substantially higher numbers and rates of pulmonary metastases. With the observed increased metastasis in Rip2-deficient mice, we examined tumors for features of EMT and demonstrated cadherin switching by loss of E-cadherin expression and gain of N-cadherin expression at the tumor periphery. We also showed increased tumoral expression of EMT transcription factors including *zeb-1*, *zeb-2*, and *snail*. NF-κB has been implicated in enhancing EMT, so the decreased activation in Rip2-deficient animals suggests an NF-κB-independent mechanism [38]. We found elevated levels of TIMP-1 in Rip2-deficient animals, which has been implicated in inducing expression of *slug*, *twist*, zeb1 and zeb2 [33]. A recent report describing down regulation of E-cadherin expression by Rip2 knockdown *in vitro* supports our findings [39]. These data support a local-regional shift in the tumor microenvironment enhanced by loss of Rip2 to facilitate EMT.

In humans, mutations in Nod2 are associated with Crohn's disease, while mutations in the Rip2 locus are linked with systemic lupus erythematosus [18], [19]. These inflammatory diseases have been associated with susceptibility to cellular stress signals as well as pathogens in the gut flora. Our findings showing increased MDSCs in the absence of Rip2 appear seemingly contradictory. However, the development of MDSCs is not clear. Potentially, the activation of tumor associated MDSCs may be triggered in the context of endogenous ligands exposed during tumorigenesis. These contextual signals may differ from other endogenous or pathogen-related signals that bias immune activation. Endogenous ligands expressed either from normal cells or specific to transformed cells that mediate these contextual signals for NLRs have yet to be identified and remain the subject of future investigations.

In the absence of Rip2, pathologic conditions such as the development of cancer, lead to microenvironment alterations and intrinsic activation of myeloid progenitors that expand the granulocytic MDSC population. These changes may influence EMT and development of metastases. Current therapeutic strategies have targeted negative T cell regulators. This includes utilizing anti-CTLA4 and anti-PD1 antibody therapies, or targeting MDSCs by inhibition of CSF-1R. The use of PRR modulation may be a novel strategy to program the TIL to favor anti-tumor immunity. This may be adopted on its own, or in conjunction with antigen-specific targeted therapy. The differences we have observed within the tumor microenvironment in our models of various PRR signaling mediator knockouts suggest that modulation of PRRs may need to be tailored to specific diseases and disease states. Our data suggest that TILs can by further shaped by activation of NLRs and function as immune modulators or adjuvant therapies.

In summary, we provide the initial evidence that intracellular NLRs represented by Rip2 can program the immune microenvironment and influence tumor invasion and metastasis in a bladder cancer model. We support a novel role of Rip2 in the development and recruitment of granulocytic MDSCs and highlight the contribution of MDSCs to the development of metastases in bladder cancer. Further study will be needed to clarify the contextual signals that trigger NLRs to mediate these functions. This study adds credence to our hypothesis that NLRs are critical sensors for the programming of the tumor immune microenvironment.
